# Iron in Neurodegeneration – Cause or Consequence?

**DOI:** 10.3389/fnins.2019.00180

**Published:** 2019-03-01

**Authors:** Alain Ndayisaba, Christine Kaindlstorfer, Gregor K. Wenning

**Affiliations:** Department of Neurology, Medical University of Innsbruck, Innsbruck, Austria

**Keywords:** iron, neurodegeneration, neurodegenerative mechanisms, mitochondrial dysfunction, autophagic-lysosomal dysfunction, protein aggregation, neuroinflammation

## Abstract

Iron dyshomeostasis can cause neuronal damage to iron-sensitive brain regions. Neurodegeneration with brain iron accumulation reflects a group of disorders caused by iron overload in the basal ganglia. High iron levels and iron related pathogenic triggers have also been implicated in sporadic neurodegenerative diseases including Alzheimer’s disease (AD), Parkinson’s disease (PD), and multiple system atrophy (MSA). Iron-induced dyshomeostasis within vulnerable brain regions is still insufficiently understood. Here, we summarize the modes of action by which iron might act as primary or secondary disease trigger in neurodegenerative disorders. In addition, available treatment options targeting brain iron dysregulation and the use of iron as biomarker in prodromal stages are critically discussed to address the question of cause or consequence.

## Introduction

Iron is involved in an abundant number of cellular processes in the brain including mitochondrial respiration, myelin synthesis, DNA synthesis, oxygen transportation, neurotransmitter synthesis and cellular metabolism (Stankiewicz and Brass, 2009; Ward et al., 2014). In the CNS, iron is present in neurons, oligodendrocytes, astroglia and microglia cells.

Within the cell, iron mediates essential functions due to its capability to participate in reaction of electron transfer thereby switching between two states: ferrous (II) and ferric (III) iron. This mechanism represents a double-edge sword because distinct levels of reactive oxygen species (ROS) are produced during these reactions enabling calcium-mediated basal synaptic transmission and long-term potentiation (Muñoz et al., 2011), but on the other hand may catalyze the so-called Fenton reaction, which generates ROS in high amounts detrimental for cellular function and eventually survival (Halliwell, 2006). Thus, tight regulation of intracellular iron homeostasis is required which is facilitated by sequestration of iron into iron-binding proteins like heme, ferritin, neuromelanin, and iron-sulfur clusters among others (Kruszewski, 2003).

Iron levels in the brain and body increase sharply up to 30 years of age due to a metabolic need during the growth process and remain stable during adulthood (Bolognin et al., 2009; Stankiewicz and Brass, 2009; Apostolakis and Kypraiou, 2017). In the aging brain however, region-specific increase of total iron is observed, probably triggered by inflammation, increased blood-brain barrier (BBB) permeability, redistribution of iron within the brain, and changes in iron homeostasis (Conde and Streit, 2006; Farrall and Wardlaw, 2009; Ward et al., 2014) and shows highest iron levels in the basal ganglia. In addition, iron accumulation varies among brain cell types, as neurons, micro- and astroglia accumulate iron over their lifespan, whereas oligodendroglial iron levels remain stable (Connor et al., 1990). Changes in regional iron distribution have been demonstrated consistently in neurodegenerative diseases via magnetic resonance imaging (MRI) (Duyn, 2010) and at post-mortem examinations (Hare et al., 2012) and since aging represents the number one risk factor for neurodegeneration, there may be a link between age-related iron accumulation and neurodegeneration (Zecca et al., 2001, 2004; Ward et al., 2014).

Alzheimer’s disease (AD), Parkinson’s disease (PD), multiple system atrophy (MSA), dementia with Lewy bodies, amyotrophic lateral sclerosis, Huntington’s disease (HD), frontotemporal dementia, corticobasal degeneration, and progressive supranuclear palsy (PSP) are primarily characterized by the deposition of insoluble protein aggregates which colocalize with iron (Coffey et al., 1989; Berg and Hochstrasser, 2006; Muller and Leavitt, 2014; Wang et al., 2016; Fernández et al., 2017; Lee et al., 2017; Sheelakumari et al., 2017; Kaindlstorfer et al., 2018; Lane et al., 2018; Moreau et al., 2018), suggesting a link between those clinically and pathologically distinct disease entities. This raises the question whether iron dyshomeostasis represents a critical factor in initiating neurodegeneration, whether it contributes to acceleration of widespread pathology as a result of nerve cell death and the consecutive release of intracellular components or whether neurodegeneration and iron accumulation constitute two completely unrelated events appearing in parallel.

In this review we aim to outline the potential links between pathophysiological mechanisms and the role of iron in neurodegeneration.

## Brain Iron Metabolism in Health and Aging

Both excess and deficiency of iron lead to impaired brain function, thus tight regulation of iron metabolism is critical.

Iron enters the brain either bound to transferrin, thereby crossing the BBB or the blood-cerebrospinal fluid (CSF) barrier (Moos and Morgan, 2000), or possibly unbound, especially in conditions that result in iron overload as transferrin becomes saturated with iron. The exact uptake mechanisms in the latter case are unknown, but it is hypothesized that free serum iron might be reduced by cellular reductants such as ascorbate (Lane and Lawen, 2008; Lane et al., 2010; Lane and Richardson, 2014) to then be imported by divalent metal transporter 1 (DMT1) or ZRT/IRT-like proteins (ZIPs) like ZIP14 or ZIP8 (Jenkitkasemwong et al., 2012). Iron uptake into astroglia is thought to be mediated mainly by non-Tf bound iron (Ashraf et al., 2018), as TfR1 has so far only been reported *in vitro* (Pelizzoni et al., 2013). DMT1, a transporter located in cellular and endosomal membranes, is found in astrocytes and is believed to facilitate transport of non-transferrin bound iron into the cytoplasm (Xu and Ling, 1994). How iron is exported from the endothelial cells is still elusive (Ward et al., 2014). Within the CSF, iron occurs mainly as holo-transferrin (two ferric iron atoms bound to apo-transferrin) that interacts with TfR1. Neurons internalize the Tf-TfR1 complex into endosomes, where iron is separated from transferrin after acidification, converted into its ferrous form via reductase STEAP3 (Ohgami et al., 2005) and transported into the cytoplasm via DMT1 (Moos and Morgan, 2004).

Iron, prone to contribute to oxidative stress, can be (i) stored within ferritin (Zecca et al., 2004), (ii) imported into mitochondria, probably via so-called mitoferrins and TfR2 (Mastroberardino et al., 2009; Horowitz and Greenamyre, 2010), to enable biosynthesis of heme and iron-sulfur clusters and contribute in the respiratory chain reaction, or (iii) be released from the cell via ferroportin 1 (Ward et al., 2014). Intracellular iron homeostasis is tightly modulated by the iron regulatory protein (IRP) and iron-responsive element (IRE) signaling pathways (Pantopoulos, 2004; Zhang D.L. et al., 2014). IRP1 and IRP2 are two RNA-binding proteins that interact with IREs, non-coding sequences of messenger RNA (mRNA) transcripts to alter translation of ferritin, ferroportin and TfR mRNA. Ferritin H and L subunits or ferroportin mRNA transcripts carry IREs within the 5′-untranslated region (UTR), whereas mRNA transcripts for TfR and DMT-1 carry IRE motifs at the 3′-UTR. Cytosolic iron binds to IRPs and induces a conformational change within the molecule that does not allow attachment to IREs. Decreased iron levels on the other hand facilitate IRP–IRE interaction: IRP binding at the 5′-UTR inhibits further mRNA translation of ferritin subunits and ferroportin; at the 3′-UTR, binding protects against endonuclease cleavage (Pantopoulos, 2004; Zhou and Tan, 2017). Ferritin represents the dominant iron storage protein in the CNS, mostly found in glia and also within neurons, whereas neuromelanin (NM) captures large amounts of iron in certain neuronal populations for longer-term storage (Zucca et al., 2017). Recent studies have demonstrated that human poly-(rC)-binding proteins 1–4 (PCBPs 1–4) are implicated in iron transfer to ferritin (Philpott, 2012; Leidgens et al., 2013; Frey et al., 2014; Yanatori et al., 2014), which is a 24 subunit heteropolymer with heavy chains (H-type ferritin) with ferroxidase activity and light chains (L-type ferritin) crucial for subsequent iron storage. H-type ferritin occurs more abundantly in neurons for rapid mobilization and use, whereas in astro- and microglia L-type ferritin predominates for iron storage. In oligodendrocytes, both forms of ferritin are expressed (Ashraf et al., 2018). Neuromelanin (NM), a dark brown pigment, is present in dopaminergic neurons of the substantia nigra, the noradrenergic neurons of locus coeruleus, the ventral tegmental area, the ventral reticular formation and the nucleus of the solitary tract in the medulla oblongata (Zecca et al., 2004; Fedorow et al., 2005), but it has also been detected in the putamen, premotor cortex and cerebellum in lower amounts (Zecca et al., 2008; Engelen et al., 2012). Ferritin degradation by the autophagy-lysosome system (Asano et al., 2011) initiates iron release which can then be reutilized or exported, mainly through ferroportin 1 (Biasiotto et al., 2016). This requires ferroxidases ceruloplasmin and hephaestin to oxidize iron for export (Hentze et al., 2004). In addition, heme-oxygenase 1 represents a stress protein which degrades heme to ferrous iron in order to maintain iron homeostasis (Nitti et al., 2018). Systemic ferroportin levels are regulated by circulating hepcidin, the main iron regulatory hormone in the body – during iron overload and inflammation, hepcidin induces ferroportin internalization and degradation (Wang and Pantopoulos, 2011). The origin of hepcidin within the brain is unknown, It may be locally produced or systemically derived by passing the BBB (Vela, 2018). Conditional ferroportin knock-out mice for example do not show any significant intracellular iron accumulation in the brain, nor do they show behavioral or histological deficits compared to wildtype mice (Matak et al., 2016), suggesting that other cellular iron export mechanisms exist.

Iron accumulates as a function of the aging brain and thereby the levels of labile, potentially harmful iron increase (Ward et al., 2014). Iron accumulating at toxic levels within neurons, as seen in neurodegeneration, may lead to cell death via apoptosis, autophagy, necrosis or ferroptosis, a recently discovered mechanism of iron-mediated cell death distinct from apoptosis (Dixon et al., 2012). In glial cells however, iron accumulation triggers the release of pro-inflammatory cytokines, thereby creating a pro-inflammatory environment (Williams et al., 2012) which promotes neurodegeneration (Xu et al., 2012).

The underlying mechanisms of age and disease related region and cell specific differences in iron levels have not been fully elucidated yet, but the distinct iron richness of the basal ganglia may explain its increased vulnerability to neurodegeneration (Hallgren and Sourander, 1958; Zecca et al., 2004; Xu et al., 2012). In neurons of substantia nigra and locus coeruleus, premotor cortex, putamen, and cerebellum, neuromelanin levels are increased for sequestration of iron, however neuromelanin may itself exhibit toxic functions which are further explained in later sections (Zecca et al., 2004, 2008).

Astro- and microglia exhibit increased iron deposition and ferritin concentrations throughout their lifespan. In contrast, oligodendrocytes that constitute the main iron reservoir in the CNS (Connor et al., 1990; Kaindlstorfer et al., 2018) do not accumulate iron by aging. Iron is essential in the development of oligodendrocytes to allow proper axon myelination, as iron deficiency induces hypomyelination that persists even after the iron imbalance has been corrected (Todorich et al., 2009). The role of iron in oligodendrocytes is further exemplified by Tim-2, a receptor selective for H-ferritin uptake (Todorich et al., 2008). Neuroinflammation in the elderly brain is induced via activated microglia, a lot of which are ferritin-positive (Kaneko et al., 1989). Of those, most exhibit a dystrophic-type morphology, and it has been suggested that iron uptake into the activated microglia cells may represent another source of toxic iron, as seen in neurodegenerative diseases (Rathnasamy et al., 2013; McCarthy et al., 2018; Refolo et al., 2018).

## Iron in Neurodegenerative Diseases – a One-Size-Fits-All?

**FIGURE 1:**
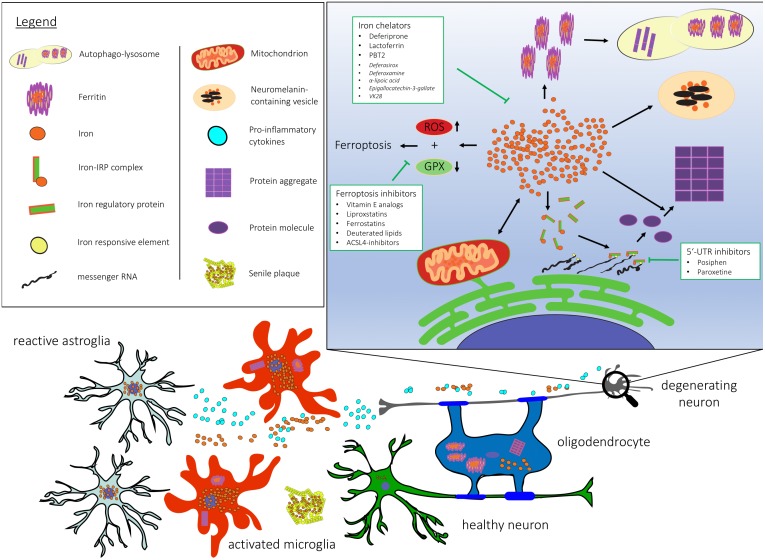
Cellular and subcellular dysregulation linked with brain iron accumulation. 5′-UTR, 5′ untranslated region; ACSL-4, Acyl-CoA Synthetase Long Chain Family Member 4; GPX, gluthathione peroxidase; RNA, ribonucleic acid; ROS, reactive oxygen species.

Iron accumulation as a hallmark feature has been confirmed in many neurodegenerative diseases. Figure 1 depicts dysfunctional pathways colocalizing with iron accumulation in neurodegeneration. Here, we review the contribution of iron to key mechanisms in neurodegeneration and evaluate its impact on pathogenesis.

### Iron and Mitochondrial Dysfunction

Mitochondria provide neurons and glia with energy in the form of ATP via oxidative phosphorylation of glucose. Functional disruption of this pathway is likely to contribute to neurodegeneration. In PD, environmental toxins that disrupt mitochondrial complex I function like rotenone induce parkinsonism (Talpade et al., 2000; Dauer and Przedborski, 2003). Also, genetic causes of early-onset PD including PARK2, PINK1, and DJ-1 may interfere with mitochondrial autophagy, thus leading to mitochondrial dysfunction (Batlevi and La Spada, 2011). Moreover, transcriptional dysregulation of coactivator PGC-1α and PPAR transcription factors, including PPAR-δ (Dickey et al., 2016) may play an important role in sporadic AD (Katsouri et al., 2016), PD, and HD. Vulnerability of neural tissue to mitochondrial dysfunction is further demonstrated in single-gene mutations causative of systemic defects in mitochondrial quality control, that can trigger neurodegeneration without having an effect on peripheral tissue (Batlevi and La Spada, 2011). In an iPSC-model of familial PD, accumulation of oxidized dopamine as an effect of mitochondrial oxidative stress has downstream effects on lysosomal function and α-synuclein (α-syn) aggregation (Burbulla et al., 2017). Inversely, lysosomal dysfunction resulting in insufficient mitophagy shows that dysfunction of one system inevitably impairs the other (Plotegher and Duchen, 2017). A large part of intracellular iron is utilized for synthesis of heme and iron-sulfur clusters in mitochondria (Singh et al., 2014). Thus, iron homeostasis is heavily dependent upon proper mitochondrial function.

Neurodegeneration with brain iron accumulation comprises a number of pediatric and adult neurological diseases characterized by parkinsonism and dementia, and excessive iron accumulation in the basal ganglia among other regions (Hogarth, 2015). Distinct genetic causes have been described within this disease spectrum, yet no direct link between neurodegeneration and iron accumulation can be spanned in most forms. Mutations in PANK2, a gene implicated in coenzyme A biosynthesis, lipids synthesis and citric acid cycle (Zhou et al., 2001), results in panthoten kinase-associated neurodegeneration (PKAN). Though this mutation may not affect iron homeostasis directly, subsequent iron accumulation suggests that mitochondrial dysfunction may trigger iron dyshomeostasis. In iPSC-derived neurons from a PKAN patient, early disease stage phenotype is reflected by mitochondrial deficiency but absent iron dysregulation and iron chelation treatment causes further exacerbation of cellular dysfunction through iron deficiency (Arber et al., 2017).

In Friedreich’s ataxia, expansions of unstable nucleotide repeats in the FXN gene result in reduced expression of frataxin (Campuzano et al., 1996), a protein thought to act as molecular chaperone in iron-sulfur clusters and heme biosynthesis as well as iron storage site (Alsina et al., 2018). Thus, lack of frataxin leads to mitochondrial iron overload and generation of ROS, which in turn impairs mitochondrial function creating a vicious cycle (Horowitz and Greenamyre, 2010). Decreased levels of frataxin were also observed in HD human and mouse brains, together with an increase of iron uptake protein mitoferrin 2 and mitochondrial iron accumulation (Agrawal et al., 2018). Interestingly, neonatal iron supplementation increases mitochondrial iron accumulation and mitochondrial dysfunction exacerbating neurodegeneration in HD mice. From this dataset it is difficult to conclude whether early-life iron exposure may cause mitochondrial dysfunction as an initiating step of neurodegeneration. It has been discussed that iron fortified infant formula within a critical window of susceptibility to iron overload may contribute to neurodegeneration in later life (Hare et al., 2015). Still, this remains highly speculative without evidence from prospective studies, but might indicate a causal role of iron in mitochondrial dysfunction. In a *Drosophila* model of Friedreich’s ataxia, upregulation of mitoferrin associated with frataxin deficiency is observed. Interestingly, targeted downregulation of mitoferrin ameliorates the phenotype while worsening overall life span, indicating that due to its physiological function dysregulation of mitoferrin has detrimental consequences (Navarro et al., 2015). Knockdown of mitoferrin-1 in an Alzheimer model of *Caenorhabditis* elegans reduces paralysis rate and slows progression of AD by decreasing mitochondrial iron accumulation and ROS generation (Huang et al., 2018), showing that mitochondrial iron homeostasis may substantially impact neurodegeneration.

Another aspect favoring a causal role of iron in mitochondrial dysfunction is that damage of mitochondrial DNA due to oxidative stress is detected consistently at an early stage of neurodegeneration, and its vulnerability can be explained by a lack of protective histones (Shokolenko et al., 2009). Dysfunctional mitochondria produce less heme and ISCs, which causes activation of IRPs and further iron accumulation leading to positive feedback loop (Mena et al., 2015). In nigral dopaminergic neurons of human PD and the rotenone mouse model, transferrin accumulates intracellularly and large parts locate in mitochondria, which may be explained by the expression of TfR2 in the mitochondrial membrane of substantia nigra dopaminergic neurons (Mastroberardino et al., 2009). Elevated ROS levels in mitochondria oxidize holo-transferrin, which then tends to release ferrous, redox-reactive iron (Mastroberardino et al., 2009). Treating rats and monkeys with rotenone leads to downregulation of TfR1 in the presence of iron accumulation, whereas TfR2 levels remain elevated (Mastroberardino et al., 2009), supporting previous findings on TfR2 stability during iron overload in hepatic tissue (Fleming et al., 2000; Mifuji et al., 2006). TfR2 stability in conditions of neurodegeneration might thus exacerbate mitochondrial dysfunction, which in turn further affects iron handling.

### Iron, Protein Misfolding, and Aggregation

Neurodegenerative diseases are characterized by extra- and/or intracellular accumulation of abnormally folded proteins and selective regional cell death in a progressive and predictable fashion (Fu et al., 2018). This includes AD, PD, MSA, dementia with Lewy bodies, PSP, frontotemporal dementia, corticobasal degeneration, HD, amyotrophic lateral sclerosis, spinocerebellar ataxias, chronic traumatic encephalopathy, and prion diseases (Fu et al., 2018). These disease entities are distinguished by clinical symptoms reflecting areas of the brain and cell types affected, by prevalence rates, risk factors and genes involved. The underlying mechanisms of protein aggregation are believed to include β-sheet-rich structures that aggregate into small oligomers and large fibrillar inclusions. There is increasing evidence that the misfolding and aggregation of proteins follows the seeding-nucleation model (Jarrett and Lansbury, 1993), where preformed seeds recruit endogenous native proteins into growing aggregates, which may then be fragmented into smaller oligomers again that are capable to spread between cells. This concept is being more and more accepted within the neuroscience community. Yet, it does not explain initial mechanisms underlying misfolding of distinct disease-associated proteins in sporadic neurodegeneration. Here, we summarize the current knowledge of interactions between iron and proteins implicated in neurodegenerative diseases.

#### Iron and α-Synuclein

Central to the pathogenesis of PD is the 17 kDa protein α-synuclein, which accumulates mainly into neuronal cytoplasmic inclusions termed Lewy bodies (Spillantini et al., 1997). Interestingly, α-synuclein can bind both ferrous and ferric iron (Peng et al., 2010), however only latter has been shown to promote aggregation (Levin et al., 2011). Phosphorylated α-synuclein at residue S129 is associated with Lewy bodies, and even though its role in PD pathogenesis has not been fully elucidated yet, it has been shown that phosphorylated synuclein exhibits increased binding affinity toward ferrous iron at the c-terminus (Lu et al., 2011). Iron and free radical generators like dopamine are able to induce aggregation of the wildtype protein in an *in vitro* model, and this effect is exacerbated by the cooccurrence of α-synuclein mutations A53T and A30P, suggesting a detrimental interplay in genetic forms of PD (Ostrerova-Golts et al., 2000). Importantly, α-synuclein possesses and IRE and IRP complexes during iron overload increase its translation resulting in elevated total α-synuclein burden (Friedlich et al., 2007; Zhou and Tan, 2017). On the other hand, it has been reported that α-synuclein exhibits ferrireductase activity that is increased in point mutations (A53T, A30P, or E46K), which may result in altered iron uptake and metabolism in the presence of divalent metal transporter 1 (Davies et al., 2011). In *Saccharomyces cerevisiae* and *Caenorhabditis elegans* models of Parkinson’s disease, expression of α-synuclein may phenocopy a high iron condition by inhibiting recycling of iron transporter ortholog of ferroportin or membrane-bound ortholog of ceruloplasmin from endocytic vesicles back to the cell membrane. Iron chelation with desferoxamine partially rescued dopaminergic neurotoxicity in *Caenorhabditis elegans* (Patel et al., 2018).

Taken together, there seems to be an intricate interplay between α-synuclein and iron, both of which promote disease progression in synucleinopathies synergistically.

#### Iron and Aβ

More than half a decade ago Goodman provided first evidence for iron accumulation in senile plaques of post-mortem AD brains (Goodman, 1953), and more recently, brain imaging of patients with early stage AD demonstrated increased iron concentrations co-localizing with Aβ plaques which may both promote disease development and progression.

Long-term treatment of APP mice with 3,5,5-trimethyhexanoyl ferrocene reveal accelerated plaque formation and more senile morphology, microglial iron inclusions and increased amounts of Aβ (Peters et al., 2018). Although the redox state of iron located in plaques is still elusive, recent studies detected iron oxide magnetite nanoparticles in the core of senile plaques (Plascencia-Villa et al., 2016; Everett et al., 2018). Furthermore, Telling et al. (2017) provide evidence for a direct iron–amyloid interaction within senile plaques. Increased iron levels are believed to enhance Aβ production via downregulation of furin, a proprotein convertase participating in α-secretase-dependent APP processing, which in turn activates β-secretase implicated in Aβ generation (Silvestri and Camaschella, 2008). SH5SY cells exposed to ferric ammonium citrate confirm an increase in β-secretase levels coupled with enhanced Aβ_42_ production, in addition iron treatment promotes accumulation of APP (Banerjee et al., 2014). Interestingly however, in a recent study ferric iron treatment in rat cortical neurons facilitates non-amyloidogenic processing of APP via α-secretase, intracellular localization of sAPPα impaired enzymatic activity of β-secretase, and thus decreases levels of sAPPβ and Aβ (Chen et al., 2018). Boopathi and Kolandaivel (2016) show that ferrous iron induces a structural conformation in Aβ toward a β-sheet structure which tends to form oligomers and fibrils, whereas other groups demonstrate that ferric iron promotes aggregation of both Aβ_40_ and Aβ_42_ as well as cytotoxicity *in vitro* (Tahmasebinia and Emadi, 2017; Galante et al., 2018). Aβ_42_ promotes magnetite nanoparticle formation (Tahirbegi et al., 2016), thus coming full circle and providing evidence for a causative role of iron in AD pathogenesis. The IRP-IRE signaling pathway is also involved in proteostasis of APP (Rogers et al., 2008). Thus, sustained iron overload may result in elevated Aβ-levels, the core constituent of extracellular plaques in AD. Intriguingly, genetic manipulation of iron metabolism substantially influences Aβ toxicity in model organisms. Overexpression of ferritin heavy chain, but also iron chelation, rescue Aβ-mediated toxicity in a Drosophila model expressing Aβ (Rival et al., 2009; Liu et al., 2011).

#### Iron and Tau

Tauopathies including AD, PSP, corticobasal degeneration, frontotemporal dementia, but also PD and HD, are characterized by neurofibrillary tangles, the core of which consists of paired helical filaments composed of hyperphosphorylated tau. Tau facilitates microtubule stabilization and regulation, regulation of axonal transport (Magnani et al., 2007; Dixit et al., 2008) and likely plays a role in neurotransmission and iron metabolism by the trafficking of APP to the cell surface (Lei et al., 2012).

These functions may be disrupted following hyperphosphorylation of tau, and iron has been shown to mediate this modification depending on its redox state. Whereas Fe (II) forms a reversible interaction through threonine residues, Fe (III) induces an irreversible conformational change, both of which promote aggregation of tau (Ahmadi et al., 2017). Hyperphosphorylation of tau, one of the key steps toward aggregation, may be induced by iron via (CDK25)/P25 complex and GSK-3β (Xie et al., 2012; Guo et al., 2013). In addition, Fe (III) may mediate nitration of tau, which prevents microtubule stabilization and is commonly found in neurofibrillary tangles and senile plaques (Nakamura and Lipton, 2011). Tau accumulation in tangles on the other hand leads to induction of heme-oxygenase 1, an antioxidant that promotes release of the redox-active Fe (II), which releases free radicals to generate oxidative stress (Ward et al., 2014). This in turn promotes tau hyperphosphorylation and aggregation (Lane et al., 2018). Intracellular iron accumulation, a common feature in tauopathies, leads to loss of tau function, which prevents iron export through impaired transport of APP to the cell membrane, where it stabilizes ferroportin (Wong et al., 2014) resulting in a vicious cycle of iron accumulation and tau pathology.

### Iron and Autophagic-Lysosomal Dysfunction

Autophagic-lysosomal dysfunction is a hallmark feature of neurodegenerative proteinopathies, either due to mutations directly affecting the autophagy-lysosome machinery, or secondary to aggregation of misfolded proteins. Iron dyshomeostasis may occur as a consequence of inadequate ferritinophagy, a term coined to describe the lysosomal degradation of ferritin molecules mediated via nuclear receptor co-activator 4 (NCOA4) (Mancias et al., 2014; Biasiotto et al., 2016). As both autophagy activation and inhibition have been reported in neurodegeneration, aberrant ferritinophagy may lead to iron dyshomeostasis manifesting as iron deficiency or overload, both of which exert deleterious effects on cell survival. In line with that, quantifications of ferritin levels in substantia nigra of PD brain reveal contradictory findings in the literature (Zecca et al., 2001; Koziorowski et al., 2007; Sian-Hülsmann et al., 2011; Friedman and Galazka-Friedman, 2012; Galazka-Friedman et al., 2012). NCOA4 levels, targeting ferritin heavy chain 1 for ferritin degradation, may be critical for ferritinophagy as factors such as oxidative stress regulate NCOA4 gene transcription (Sharifi et al., 2008). Pharmacological inhibition of autophagy leads to NCOA4 accumulation (Dowdle et al., 2014), probably as a compensatory mechanism. In addition, NCOA4-depletion seems to have a protective effect during oxidative stress (Mancias et al., 2014), possibly contrabalancing transcriptional and translational regulation of ferritin in situations of stress (Torti and Torti, 2002). Another part of the autophagic machinery slowly accumulating iron high amounts of iron, copper and zinc as well as various toxins over the course of several decades constitutes neuromelanin (Zecca et al., 2008). In addition, intracellular dopamine in excess can be metabolized into the more stable NM molecule. However, externalization of neuromelanin organelles and subsequent release of content as seen in PD activates microglia, which may accelerate further cell death and release of neuromelanin-containing vesicles, thereby creating a self-perpetuating cycle (Zhang W. et al., 2013; Zucca et al., 2017). Recent data suggests that neuromelanin-containing vesicles in PD may represent a specialized autolysosomal compartment, where proteins and lipids not otherwise degraded are accumulated in a very slow turnover rhythm (Zucca et al., 2018). Neuromelanin accumulates within vesicles over time, and in PD, and a strong correlation between nigral cell loss and amount of pigmented neuromelanin-immunoreactive neurons could be shown, providing first evidence that neuromelanin-pigmented cells may exhibit increased vulnerability during PD pathogenesis (Zucca et al., 2018). One possible explanation is that at a certain threshold of iron level in PD brains buffering capacities in the form of NM and ferritin are exhausted (Good et al., 1992; Jellinger et al., 1992), and then redox-active iron is freed and may have deleterious consequences and significantly contributes to subsequent pathology (Faucheux et al., 2003).

### Iron and Neuroinflammation

Microglia are in a constant cross-talk with neurons and are capable of sensing neuronal dyshomeostasis. Microglial activation is a healthy reaction against disease and injury, accompanied by the release of pro- and anti-inflammatory factors, ROS and recruitment molecules (Colton and Gilbert, 1987; Graeber et al., 1988; Hurley et al., 1999). Chronic neuroinflammation however, a hallmark feature of neurodegenerative diseases, creates a pro-apoptotic environment resulting in neuronal death and thus becomes a driver of neurodegeneration itself (Lull and Block, 2010). AD and PD brains show extensive proliferation of reactive macrophages and activated microglia. In addition to the cocktail of cytokines and ROS released by microglia cells, they also produce high levels of NAPDH oxidase and nitric oxide synthase, two main players of oxidative stress and ROS generation during inflammation (Urrutia et al., 2014).

Interestingly, microglia turn out to be far more efficient in sequestering iron compared to other brain cell types, as levels of ferritin induced by iron exposure in an organotypic slice culture model are highest in microglia and oligodendrocytes, whereas ferritin is rarely detected in astrocytes and, surprisingly, never in neurons (Healy et al., 2016).

Exposing cultured neurons to ferric iron reveals accumulation of iron, however microglia accumulates eightfold more iron than neurons and 4.7-fold more than cultured astrocytes after 24 h (Bishop et al., 2011). Activated microglia in substantia nigra of post-mortem PD brains exhibit enhanced ferritin immunoreactivity (Mirza et al., 2000), yet iron burden does not correlate with ferritin in a linear fashion, as high iron saturation in microglia may be achieved despite low ferritin baseline, as seen in conditions of acute oxidative stress (Mehlhase et al., 2005; Thomsen et al., 2015). In neurodegeneration, iron-containing microglia co-localize with affected areas (Andersen et al., 2014) and there is some intriguing evidence pointing toward a substantial contribution of microglia-associated iron in disease. Ferrous iron in primary midbrain cultures causes progressive dopaminergic neurodegeneration mediated by microglial β-nicotinamide adenine dinucleotide phosphate oxidase 2, a superoxide generator, and inhibition of the enzyme prevents neurotoxicity reflected in decreased levels of superoxide (Zhang W. et al., 2014). Intriguingly however, Wang et al. report that iron accumulation in microglia alone is not sufficient to trigger release of proinflammatory cytokines TNF-α, IL-1β, and IL-6, instead MPP+ is required to trigger enzymatic release, which is then aggravated if iron accumulation is present (Wang et al., 2013). Inflammatory stimuli including TNF-α, IL-β and toll-like receptor-4 agonist lipopolysaccharide (Yang et al., 2002) on the other hand are able to trigger neuronal iron accumulation via alteration of DMT1 and FPN mRNA levels (Urrutia et al., 2013). This is supported by another study showing increased DMT1 and TfR1 and decreased FPN1 protein levels following TNF-α or IL-1β treatment (Wang et al., 2013).

Increased iron uptake in substantia nigra in PD patients has been linked with altered expression of lactoferrin receptor on vulnerable dopaminergic neurons (Faucheux et al., 1995). In addition, lactoferrin has been implicated in AD pathogenesis due to its presence in senile plaques and neurofibrillary tangles in the limbic system (Wang et al., 2015). Lactoferrin is produced only by activated microglia (Fillebeen et al., 2001; An et al., 2009; Wang et al., 2015) and it is suggested that it may have a protective function in neurodegeneration by modulating mitochondrial calcium homeostasis (Fillebeen et al., 1999; Rousseau et al., 2013) rather than sole iron chelation following MPP+ injury (Wang et al., 2015).

Astroglia as part of the BBB are involved in strict separation and regulation of brain iron metabolism from the periphery (Moos et al., 2007; Burkhart et al., 2016). They express coeruloplasmin to facilitate iron uptake and distribution (McCarthy and Kosman, 2015) and hephaestin, indicating ferroxidase activity (Wang et al., 2007). However, astroglia contain relatively small amounts of ferritin and iron (Mirza et al., 2000; Zecca et al., 2004; Bartzokis et al., 2007), yet *in vitro* studies in cultured astrocytes show that cultured astrocytes are surprisingly resistant to ferrous iron compared to neurons and oligodendrocytes, even though intracellular iron burden is comparable (Kress et al., 2002; Oshiro et al., 2008). Possibly this is due to an extensive antioxidative system including metallothioneins (Waller et al., 2018). Astroglia possess heme oxygenase-1, which has been shown to be neuroprotective in different models of PD by impeding oxidative stress (Xu et al., 2016; Yu et al., 2016), it has to be noted however that during the degradation of heme catalyzed by the enzyme, bio-reactive iron is released to participate in Fenton’s reaction (Cuadrado and Rojo, 2008). Astroglial increase of iron levels is observed in substantia nigra of PD (Jellinger et al., 1990), which is accompanied by astroglial activation, reflected in part by the up-regulation of lipocalin-2, which, if overexpressed in experimental conditions, initiates nigrostriatal dopaminergic neurodegeneration that is aggravated following iron treatment (Kim et al., 2016). Neurotrophic factors BDNF and GDNF, produced by astroglia besides oligodendrocytes, prevent neuronal iron accumulation by affecting IRPs linked with DMT1 expression (Zhang H.Y. et al., 2014), highlighting the crucial role of astroglial health on iron regulation. In case of iron accumulation, exemplified by a study from Rathore et al. including treatment of rat astrocytes with ferrous iron, increased expression of DMT1, ferroportin and both ferritin subunits are observed. However, co-treatment with TNF-α diminished the increase in H-ferritin, whereas the elevated L-ferritin levels are not altered (Rathore et al., 2012). This raises the question whether microglial activation in form of TNF-α secretion might impair astroglial iron handling and storage. In the 6-OHDA neurotoxin rat model of PD, altered BBB permeability is associated with microglial iron accumulation and increased levels of L-ferritin besides neurodegeneration in substantia nigra (Olmedo-Díaz et al., 2017). Using Mössbauer spectrometry, Friedman et al. (2011) determine decreased levels of L-ferritin in substantia nigra in PD brains in contrast to experimental results.

Iron as cause of neuroinflammation has also been shown in ALS by introducing the G93A mutation of ALS-associated human superoxide dismutase 1 (SOD1) in mice, which results in iron accumulation of ventral motor neurons. Intriguingly, an increase in the enzymatic activity of TNF-α – converting enzyme and subsequent enhanced secretion of soluble TNF-α is observed triggered by iron whereas chelation with deferoxamine mesylate delays disease onset and prolongs overall lifespan (Milanese et al., 2014).

### Ferroptosis in Neurodegeneration

Ferroptosis represents a recently discovered form of cell death independent of the caspase pathway and involves iron dysregulation, lipid peroxidation and inflammation as major hallmarks (Dixon, 2017). Central to this phenomenon is the depletion of glutathione, an antioxidant that buffers ROS and binds to labile iron (Hider and Kong, 2011) to prevent Fenton reaction with subsequent ROS production. This imbalance in cellular redox homeostasis causes the accumulation of lipid ROS and thus lipid peroxidation (Gao et al., 2016; Abdalkader et al., 2018). Morphological changes reflecting the consequences include reduced mitochondrial volume (Dixon et al., 2012; Xie et al., 2016), reduced density of mitochondrial membranes (Xie et al., 2016), chromatin condensation (Dixon et al., 2012), cytoplasmic swelling, and eventually rupture of mitochondrial outer membrane (Friedmann Angeli et al., 2014; Xie et al., 2016) and plasma membrane (Dixon et al., 2012). A number of potent inducers of ferroptosis have been identified (Stockwell et al., 2017), among which the small molecule erastin potently induces ferroptosis via inhibition of the xCT cystine/glutamate antiporter, as cystine is essential for glutathione synthesis. Reduced gluthatione is utilized by glutathione peroxidase GPX4 (Yang et al., 2014) to detoxify phospholipid hydroperoxides and hydrogen peroxides (Ursini and Bindoli, 1987). Interestingly, GPX4 knock-out in mice revealed not only iron dysregulation, lipid peroxidation and inflammation, but in addition early signs of an AD phenotype including behavior dysfunction and hippocampal neurodegeneration (Seiler et al., 2008; Hambright et al., 2017). The deleterious effects on neuronal health and survival upon ablation of GPX4 in motor neurons might confer a role of ferroptosis to degenerative motor neuron diseases like amyotrophic lateral sclerosis (Chen et al., 2015).

Key features of ferroptosis including lipid peroxidation and reduced glutathione levels have been detected previously in the substantia nigra in PD (Dexter et al., 1989; Jenner et al., 1992) and colocalize with accumulated iron. According or Jenner et al. these findings may represent early events in the disease (Jenner et al., 1992). In addition, mitochondrial damage, commonly observed in PD as well, may be mediated by the interaction of α-synuclein with mitochondrial membranes, thus aggravating mitochondrial vulnerability and oxidative stress further (Parihar et al., 2008).

Therefore, targeting ferroptosis in neurodegeneration may represent an attractive target for disease modification. Potential candidates include vitamin E analogs, liproxstatins and ferrostatins, deuterated lipids and ACSL4 (Acyl-CoA Synthetase Long Chain Family Member 4) inhibitors (Angeli et al., 2017).

## Can Iron Cause Neurodegeneration?

Based on the literature reviewed to this point it is certain that iron contributes substantially to neurodegeneration, however so far, no compelling evidence was provided to attribute a causal role to iron, which would imply (1) detection at an early stage if not as first sign, and (2) evidence for disease modification if targeted appropriately. We aim to address both issues based on current knowledge and progress made to this point.

### Targeting Iron Dyshomeostasis for Disease Modification in Neurodegeneration

In the past few years numerous targets for treatment of iron dysregulation have been identified including reduction of iron burden but also inhibition of downstream effects. However, the numerous physiological functions of iron and defects resulting from aggressive iron depletion need to be considered. Therefore, the following features of treatment of brain iron dyshomeostasis could be taken into account: ability to pass BBB and consecutive cell membranes; chelation of excess iron with moderate affinity to avoid depletion of transferrin and iron-associated proteins; prevention of side effects due to iron removal in areas lacking iron overload (Boddaert et al., 2007; Hamilton et al., 2017; Loza-Rosas et al., 2017). Here, we will discuss potential candidates based on those selection criteria.

#### Deferiprone

Deferiprone is an iron chelator currently in use for systemic iron overload conditions like thalassemia major, but its benefit in neurodegenerative conditions lacking systemic iron dysregulation has been shown in a small trial in 9 patients with Friedreich’s ataxia, who received deferiprone for 6 months. Treatment results in decreased iron load in dentate nuclei as visualized by MRI, an area implicated in neurodegeneration. Moreover, motor skills and signs of neuropathy are improved especially in the younger patients, and no severe side effects are reported (Boddaert et al., 2007). Deferiprone acts as “conservative iron chelator” by redistributing iron from overloaded areas to areas deprived of iron, thereby keeping overall iron levels stable (Cabantchik et al., 2013). In a 12 months double-blind pilot study (FAIRPARK-I), 40 PD patients were recruited, and treatment with deferiprone reduces substantia nigra hyperechogenicity in MRI and shows clinical improvement determined by the Unified PD Rating Scale (UPDRS) (Devos et al., 2014).

Interestingly however, in another phase II trial on deferiprone 22 early-disease patients (Martin-Bastida et al., 2017) reveal no change in iron content of putamen, striatum nor substantia nigra except for 3 cases. In dentate and caudate nuclei decreased iron load is determined by MRI. In this study, no improvement in motor-UPDRS is observed, even though a trend is achieved in the high dose-tier (30 mg/kg). An ongoing phase III trial, FAIRPARK-II, will reveal further insights into the potential benefits of deferiprone in PD.

#### Lactoferrin

Lactoferrin (LF) is an iron-binding glycoprotein with 60% amino acid sequence homology to transferrin (Metz-Boutigue et al., 1984). However, LF affinity for iron is 300 times higher than transferrin (Dhennin-Duthille et al., 2000) and it can act by binding two iron, zinc, copper particles or other metals. In AD brain, abnormal LF content was described already a few decades ago (Brown et al., 1985) and in 2010 Wang et al. could show that LF is present in AD model mice, but not healthy wildtype mice (Wang et al., 2010). There is a strong correlation between LF and Aβ, as LF levels increase following generation of Aβ, and with Aβ plaque formation lactoferrin continues to accumulate (van de Looij et al., 2014).

There is evidence of a protective function of lactoferrin as exogenous application results in reduced Aβ deposition and improvement of cognitive decline in AD mice via enhanced non-amyloidogenic processing of APP and α -secretase expression and activity through ERK/1/2-CREB and HIF-1α pathways (Guo et al., 2017). In MPTP mice modeling PD, administration of LF results in reduction of MPTP-induced iron accumulation via suppressed upregulation of DMT1 and TfR. Furthermore, neuroprotection is mediated by an increase in BDNF and HIF-1α with downstream activation of ERK/1/2-CREB pathway (Xu et al., 2018). In addition, LF receptors are found at the BBB, therefore LF may also be utilized as carrier for iron chelators that would otherwise be excluded from CNS. Kamalinia et al. conjugated LF with deferasirox to inject into AD mice, which ameliorates memory function, reduces Aβ levels, and leads to attenuation of apoptosis markers and increase in autophagy (Kamalinia et al., 2013). In a recent pilot study 50 AD patients were treated with lactoferrin for 3 months to find significant improvement in antioxidant and anti-inflammatory markers in serum, and decrease in Aβ_42_, phosphorylated tau, interleukin-6 and caspase-3 as well as improved cognitive function (Mohamed et al., 2019), possibly opening the doors toward new treatments in AD, however further follow-up data are required.

#### PBT2

Metal chaperone PBT2 is a derivate of clioquinol, an iron, copper and zinc chelator. In a phase II trial for AD clioquinol improved cognition and decreased Aβ plasma levels (Ritchie et al., 2003), however in a transgenic AD mouse model toxicity manifested by myelinopathy was observed (Zhang Y.H. et al., 2013). PBT2 on the other hand improved cognition and decreased intracerebral Aβ levels in preclinical AD models (Adlard et al., 2011, 2014). A phase II trial in 78 AD patients revealed significant reduction in CSF Aβ_42_ and improved cognition by two measures of executive function (Lannfelt et al., 2008; Faux et al., 2010).

#### IRE (5′UTR) Inhibitors

Another promising target in the self-perpetuating cycle of iron and disease-associated protein accumulation represents the inhibition of the IRE in the 5′-UTR of α-synuclein and APP (Zhou and Tan, 2017). Two compounds have now been tested in clinical trials, these include Posiphen, a phenyl carbamoyl analog of (+)-physostigmine, second-generation 8-hydroxyquinoline analog metal chaperone PBT2, and paroxetine, an antidepressant of the selective serotonin-reuptake inhibitor class.

Posiphen potently inhibits translation of APP and α-synuclein in preclinical models (Shaw et al., 2001; Yu et al., 2013; Teich et al., 2018). In a phase I human trial and a small non-randomized study of five subjects with mild cognitive impairment, administration of posiphen is well tolerated and decreases levels of sAPPα, sAPPβ, total and phosphorylated tau, and inflammatory marker in CSF. In addition, a trend toward lower Aβ_42_ is observed (Maccecchini et al., 2012).

Besides, paroxetine is widely used in the treatment of depression acting through selective inhibition of serotonine reuptake and may represent a suitable candidate for drug repurposing in AD. Preclinical *in vitro* and *in vivo* evidence supports the effects of paroxetine as chemical IRE modulator on APP expression (Payton et al., 2003; Tucker et al., 2006; Bandyopadhyay and Rogers, 2014), and its clinical safety has obviously been proven due to its indication in depression.

### Iron as MRI Biomarker for Disease Progression

One of the major drawbacks in therapeutic research of neurodegenerative diseases is a lack of biomarkers for earlier diagnosis, providing a chance for successful disease-modifying treatments. Advances in MRI technology to facilitate more accurate and specific detection of iron may help to determine the biomarker status of iron accumulation at preclinical stages of neurodegeneration. For an extensive review on the use of MRI for brain iron detection we refer to excellent reviews (Schweser et al., 2011; Schipper, 2012).

In premotor PD patients, MRI scans revealed increased echogenicity in substantia nigra, probably indicative of labile iron (Berg et al., 2011). De Marzi et al. (2016) investigated a cohort of patients with idiopathic rapid eye movement sleep behavior disorder (iRBD), a majority of which went on to develop PD. Interestingly, loss of dorsolateral nigral hyperintensity in a T2^∗^ map, a sign for low neuromelanin reflecting the loss of dopaminergic neurons (Blazejewska et al., 2013), was determined both in iRBD as well as in PD patients, indicating that loss of dorsolateral nigral hyperintensity may represent a promising biomarker for prodromal PD (De Marzi et al., 2016). In a cohort with carriers of the apolipoprotein 𝜀4 allele at risk for AD and patients with mild cognitive impairment, representing early stage AD, quantitative susceptibility mapping MRI revealed higher Ab plaque load as well as increased iron concentrations colocalizing with Ab plaques both in cortical as well as sub-cortical brain areas (van Bergen et al., 2016). In Huntington’s disease however, examination of premanifest gene carriers and early Huntington’s disease patients exhibit no differences in iron content in asymptomatic carriers, whereas in early patients a lower R2^∗^ parameter value, indicating alterations in iron burden, was observed. In this case, iron measurement by MRI may rather be a marker of disease progression (Di Paola et al., 2014). In genetically and phenotypically distinct disorders like ALS with variable upper and lower motor neuron affection, a reliable biomarker is urgently needed for both early and accurate diagnosis. MRI in ALS patient cohorts shows hypointensity in deep layers of motor cortex, and increased hypointensity correlates with more severe affection of upper motor neurons (Kwan et al., 2012). Direct comparison of MRI signal changes in ALS with histological examination of post-mortem ALS brains revealed that motor cortex hypointensity corresponds to microglial iron accumulation, which may have utility as marker for disease progression (Pallebage-Gamarallage et al., 2018). Taken together, brain iron detection via MRI will be a powerful tool in the future both for early diagnosis and for evaluation of disease progression in neurodegenerative disorders. Detection of biochemical or structural components implicated in the disease process in addition to brain iron have immense potential to elucidate early pathophysiological changes in the otherwise inaccessible brain.

## Conclusion and Outlook

Experimental data show that many neurodegenerative diseases represent conditions of multifactorial cellular dysfunction associated with iron dyshomeostasis present already at early disease stages, yet preclinical models may not be able to answer what comes first in sporadic neurodegenerative diseases. Intriguingly however, iron substantially contributes to and drives many aspects of neurodegeneration, plus, genetic modification of iron metabolism resulted in rescue of neurodegeneration in different animal models, indicating a causal role of iron in neurodegenerative disorders. Advances in MRI technology to facilitate more accurate and specific detection of iron hold great promise to implement iron as a biomarker for preclinical stages of neurodegeneration, probably a crucial treatment window for upcoming therapies. Moreover, CSF levels of iron related proteins in sporadic neurodegenerative diseases may aid in early diagnosis in the future, like CSF ferritin in AD (Ayton et al., 2015, 2017). Currently, a number of various promising compounds targeting different aspects of iron dysregulation besides conventional iron chelation are being tested at different clinical and preclinical trial stages. Recently discovered mechanisms like ferroptosis may pose promising targets for disease modification via ferrostatins in the future.

## Author Contributions

AN wrote the manuscript. CK and GW edited and revised the manuscript.

## Conflict of Interest Statement

CK and GW are employed by the Medical University of Innsbruck, Austria. The remaining author declares that the research was conducted in the absence of any commercial or financial relationships that could be construed as a potential conflict of interest.
